# Comparative evaluation of three high-molecular-weight DNA extraction kits for Oxford Nanopore sequencing of *Clostridioides difficile* and *Clostridium perfringens*

**DOI:** 10.1128/spectrum.02407-26

**Published:** 2026-09-11

**Authors:** Alexandre Mrozinski, Daniel Martak, Guillaume Reboul, Muriel Ehmig, Laure Diancourt, Frédéric Barbut, Caroline Le Maréchal, Edward Topp

**Affiliations:** 1 Agroécologie Laboratory, INRAE, Université Bourgogne Europe 27011, Dijon, France; 2 ANSES, Laboratoire de Ploufragan-Plouzané-Niort, Unité HQPAP, BP53, Ploufragan, France; 3 National Reference Laboratory for Clostridioides difficile Saint-Antoine Hospital, AP-HP 26930 , Paris, France; 4 National Reference Laboratory for Anaerobic Bacteria and Botulism, Institut Pasteur 27058, Paris, France; 5 INSERM 1139, FPRM, Université Paris Cité 555089, Paris, France; 6 Department of Biology, University of Western Ontario 98604, London, Ontario, Canada; University of Mississippi, University, Mississippi, USA

**Keywords:** *Clostridioides difficile*, *Clostridium perfringens*, whole-genome sequencing, Oxford Nanopore, MinION, long-read sequencing, plasmid, toxin-encoding genes

## Abstract

*Clostridioides difficile* and *Clostridium perfringens* are Gram-positive, spore-forming anaerobic pathogens affecting humans and animals, for which genomic data have been mainly generated using short-read or hybrid sequencing approaches. In this study, we evaluated three commercial non-bead-beating DNA extraction kits designed for high-molecular-weight DNA recovery for Oxford Nanopore long-read whole-genome sequencing of two *C. difficile* and two *C. perfringens* strains, including one reference strain and one clinical or environmental isolate per species. Based on sequencing performance and kit ease of use, one kit was selected for additional sequencing of plasmid-carrying strains of both species. All three kits allowed correct identification of sequence types, toxin-encoding genes, and antimicrobial resistance determinants, confirming their suitability for clinical and epidemiological applications. However, the BT MasterPure Kit provided the highest DNA concentrations, longest fragment sizes, and superior read lengths and N50 values, particularly for *C. difficile*, achieving >100× coverage and enabling reliable circularization of chromosomes and plasmids, including a *C. difficile* metronidazole resistance plasmid and *C. perfringens* plasmids carrying toxin and antibiotic resistance genes. The other kits produced slightly lower DNA yields, resulting in shorter reads and reduced genome coverage for *C. difficile*, highlighting the challenge of extracting high-quality DNA from Gram-positive, spore-forming bacteria. Overall, this study provides practical guidance for selecting DNA extraction protocols optimized for Oxford Nanopore sequencing of *C. difficile* and *C. perfringens*, supporting high-quality genome assemblies and plasmid characterization and facilitating the routine genomic surveillance of clinically relevant spore-forming pathogens.

**IMPORTANCE:**

High-quality genomic data are essential for accurate characterization of *Clostridioides difficile* and *Clostridium perfringens*, two clinically and epidemiologically important Gram-positive, spore-forming pathogens. However, long-read sequencing performance can be strongly influenced by the choice of DNA extraction method, particularly for organisms with robust cell walls, where commonly used methods can lead to fragmented DNA. In this work, DNA of four strains was extracted using three commercial high-molecular-weight DNA extraction kits and sequenced using Oxford Nanopore Technologies. The best-performing kit was also evaluated using three additional strains known to harbor plasmids in order to assess its plasmid recovery efficiency. The results demonstrated successful plasmid recovery, circularization, and characterization. DNA extraction protocols optimized for Oxford Nanopore sequencing enable the rapid and cost-effective characterization of *C. difficile* and *C. perfringens* for genomic surveillance or outbreak investigations.

## INTRODUCTION

Clostridia are ubiquitous spore-forming, rod-shaped anaerobic bacteria. Some clostridial species can cause disease in animals and humans through the production of extracellular toxins. Some of these species pose a significant burden to human and animal health, such as *Clostridioides difficile* and *Clostridium perfringens. C. difficile* is a major cause of healthcare-associated diarrhea worldwide, responsible for antibiotic-associated diarrhea and colitis, and represents a significant public health concern due to the high rate of infection recurrence (1–3). Diarrhea associated with *C. difficile* is mainly caused by the production of three toxins: toxin A (*tcdA*), toxin B (*tcdB*), and the binary toxin (*cdtA* and *cdtB*) (4). It has been classified as an urgent threat by the U.S. Centers for Disease Control and Prevention, with more than 223,000 cases of infections and 12,800 associated deaths reported in the United States in 2017 (5). It is also involved in infections in horses, pigs, and rabbits (6). *C. perfringens* is a ubiquitous bacterium associated with foodborne toxin-mediated diseases, as well as traumatic gas gangrene, which is primarily associated with war injuries and surgical wounds (7, 8). The genes encoding toxins vary from one strain to another, which allows them to be classified into different toxinotypes. The main toxins are encoded by the genes *cpa*, *cpb*, *etx*, *itx*, *cpe*, and *netB* (7). This pathogen causes more than one million foodborne illnesses in the United States each year (9). It also has major impacts on livestock production, with economic losses in poultry due to necrotic enteritis caused by *C. perfringens* estimated at approximately US$6 billion annually worldwide (10, 11).

Although they may play a major role in pathogenicity and antimicrobial resistance, *C. difficile* plasmids have not been well characterized to date (12). Toxin genes are most often chromosomally encoded. However, in some cases, the genes encoding the binary toxin (*cdtA* and *cdtB*) and toxin B (*tcdB*) have been reported to be carried on a plasmid (13). A large plasmid conferring resistance to metronidazole, an antibiotic previously used for the treatment of *C. difficile* infections, has recently been identified (14). Plasmids also play a critical role in the pathogenicity of *C. perfringens* (15). The genes encoding toxins are mainly found on plasmids, which also contain genes responsible for antimicrobial resistance (15). The enterotoxin gene (*cpe*) can be located either on the chromosome or on plasmids. Toxin genes such as the necrotic enteritis B toxin (*netB*) and the iota toxins itx (*iap* and *ibp*) are known to be exclusively associated with plasmids (7). *C. perfringens* strains can carry up to 10 plasmids, some of which can be transferred via conjugation to increase pathogenicity (16).

In the last two decades, whole-genome sequencing (WGS) has become the gold standard for bacterial typing and epidemiological investigations. The WGS of *C. difficile* and *C. perfringens* DNA has primarily been conducted using short-read technologies (e.g., Illumina Technologies) or hybrid approaches combining short- and long-read sequencing (15, 17). Although short-read sequencing is highly accurate, it is unable to resolve repetitive regions and therefore struggles to fully assemble chromosomes and plasmids. To overcome these challenges, long-read technologies have emerged. In particular, the nanopore-based sequencing approach by Oxford Nanopore Technologies (ONT) can generate long reads that enable complete genome assembly and recovery of complete plasmids (18–20). Complete genome assembly through long-read sequencing also allows better characterization of the genetic environment of resistance genes and of mobile genetic elements (MGE) (21).

The main challenge for long-read sequencing of Gram-positive bacteria is the isolation of high-molecular-weight (HMW) DNA. The thick peptidoglycan layer of Gram-positive organisms, particularly in spore-forming species such as *C. difficile*, complicates cell lysis and often necessitates mechanical disruption methods. These approaches can result in fragmented or low-quality DNA yield (22), thereby compromising read length and assembly contiguity and preventing the complete reconstruction of chromosomes and plasmids.

This study aimed to identify the DNA isolation method that produced the highest DNA yield, as well as the best read quality for *C. difficile* and *C. perfringens* using Oxford Nanopore sequencing. Specifically, we aimed to establish which kit most effectively supports complete genome assembly, including chromosome and plasmid circularization, the reliable detection of toxin-encoding genes and antibiotic resistance genes (ARGs), and the accurate identification of sequence types (STs). By comparing extraction protocols, this work provides practical guidance for optimizing long-read sequencing workflows for *C. difficile* and *C. perfringens*.

## MATERIALS AND METHODS

### Study design

The present study comprised two experiments.

#### Experiment 1

**Fig 1:**
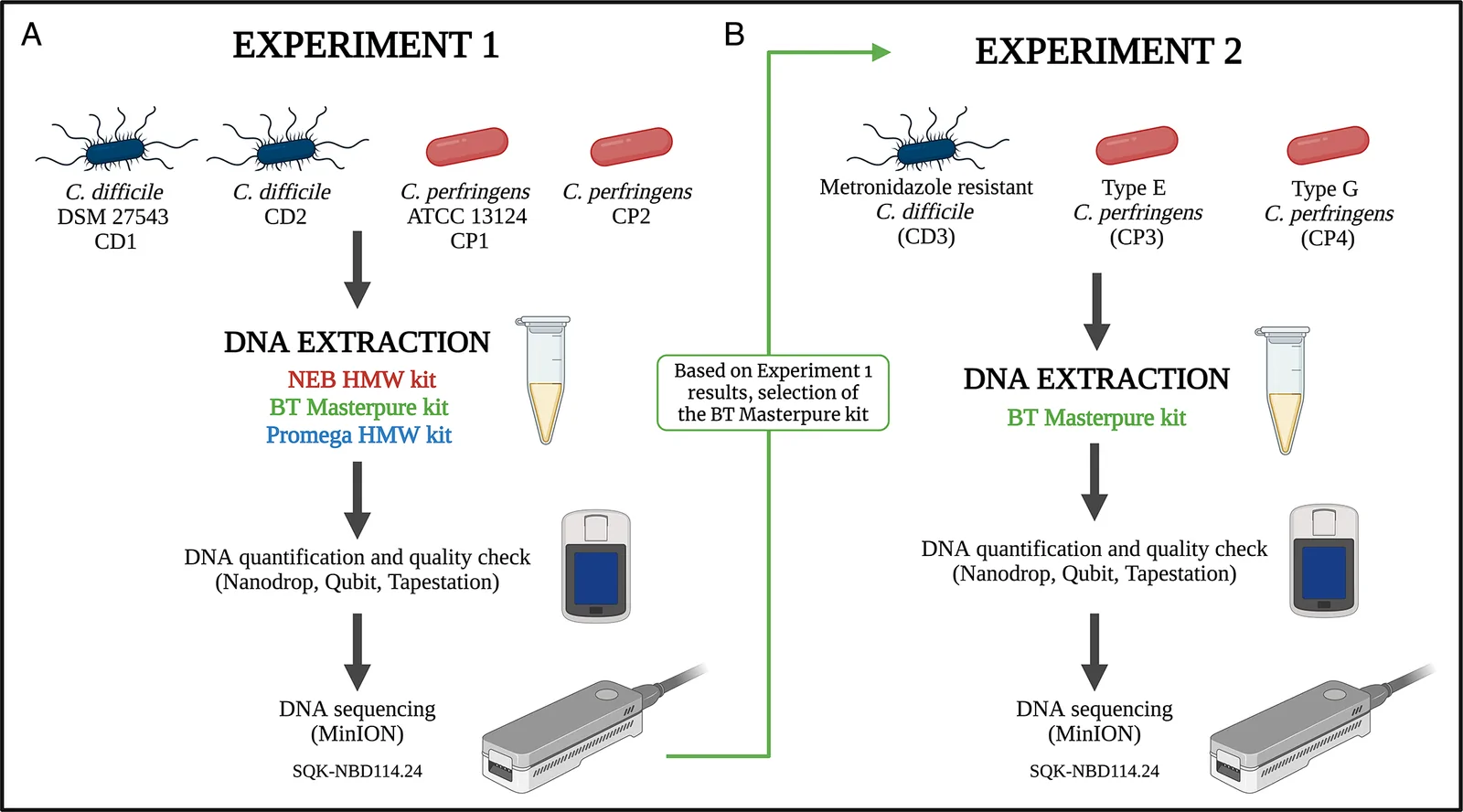
Workflow used for optimizing Nanopore sequencing of *Clostridioides difficile* and *Clostridium perfringens*. (**A**) Experiment 1: DNA of two *C. difficile* strains (CD1 and CD2) and two *C. perfringens* strains (CP1 and CP2) was extracted using three different DNA extraction kits: New England Biolabs Monarch High Molecular Weight DNA Extraction Kit for Tissue (NEB HMW), Biosearch Technologies MasterPure Complete DNA and RNA Purification Kit (BT MasterPure), and Promega High Molecular Weight DNA Extraction Kit (Promega HMW). (**B**) Experiment 2: DNA of one *C. difficile* (CD3) and two *C. perfringens* (CP3 and CP4) was extracted using only the BT MasterPure Kit. A preliminary lysozyme treatment was performed prior to DNA extraction for each kit. DNA quantity and quality were assessed using NanoDrop, Qubit (high-sensitivity and broad-range assays), and TapeStation genomic DNA analysis. Sequencing libraries were prepared using the Rapid Barcoding Kit v14 (SQK-NBD114.24) and sequenced on R10.4.1 flow cells (Oxford Nanopore Technologies). Created in BioRender (BioRender, H., 2026; https://BioRender.com/ljf0124).

To determine the most suitable DNA extraction kit for long-read sequencing of *C. difficile* and *C. perfringens*, we first sequenced two *C. difficile* and two *C. perfringens* strains using three different DNA extraction kits: the Wizard HMW DNA Extraction Kit (Promega, Charbonnières-les-Bains, France) (Promega HMW) (TM604, revised 7/22), the MasterPure Complete DNA and RNA Purification Kit (Biosearch Technologies, Hoddesdon, UK) (BT MasterPure) (protocol dated to February 2025), and the Monarch HMW DNA Extraction Kit for Tissue (New England Biolabs, Ipswich, MA, USA) (NEB HMW) (protocol dated to February 2025) (Fig. 1).

#### Experiment 2

Based on the results of experiment 1, the most effective kit was tested for its ability to recover plasmids. To this end, three additional plasmid-carrying strains were sequenced: one *C. difficile* strain and two *C. perfringens* strains (Fig. 1).

### Bacterial strains and prior characterization

In experiment 1, two reference strains, *C. difficile* DSM 27543 (CD1) (NCBI RefSeq: NZ_CP010905.2) and *C. perfringens* ATCC 13124 (CP1) (NCBI RefSeq: NC_008261.1), were selected. We also selected one *C. difficile* strain collected from sewage sludge (CD2) and one *C. perfringens* strain collected in a wastewater treatment plant effluent sample (CP2). In experiment 2, two *C. perfringens* strains (CP3 and CP4) and one *C. difficile* strain (CD3) were selected based on the presence of toxin or antimicrobial resistance genes known to be plasmid encoded. The characteristics of all strains are detailed in Table 1.

**TABLE 1:** Characteristics of the *Clostridioides difficile* and *Clostridium perfringens* strains used in this study^*a*^

Species	Label	Strain/source	Experiment	Extraction kit	Lysis buffer	Sequence type	Toxinotype	Plasmid
*C. difficile*	CD1	Reference strain (630, DSM 27,543) (NZ_CP010905.2)	1	NEB HMW	STET (90%) + TE (10%) + lysozyme 10 mg/mL	54	*tcdA*+, *tcdB*+	–
*C. difficile*		Reference strain (630, DSM 27,543) (NZ_CP010905.2)	1	BT MasterPure	TE (100%) + lysozyme 10 mg/mL	54	*tcdA*+, *tcdB*+	–
*C. difficile*		Reference strain (630, DSM 27,543) (NZ_CP010905.2)	1	Promega HMW	TE (100%) + lysozyme 10 mg/mL	54	*tcdA*+, *tcdB*+	–
*C. difficile*	CD2	Sewage sludge	1	NEB HMW	STET (90%) + TE (10%) + lysozyme 10 mg/mL	–	*tcdA*+, *tcdB*+, *cdtA*+, *cdtB*+	–
*C. difficile*		Sewage sludge	1	BT MasterPure	TE (100%) + lysozyme 10 mg/mL	–	*tcdA*+, *tcdB*+, *cdtA*+, *cdtB*+	–
*C. difficile*		Sewage sludge	1	Promega HMW	TE (100%) + lysozyme 10 mg/mL	–	*tcdA*+, *tcdB*+, *cdtA*+, *cdtB*+	–
*C. difficile*	CD3	Human, stools	2	BT MasterPure	TE (100%) + lysozyme 10 mg/mL	–	No toxin	pCD-METRO
*C. perfringens*	CP1	Reference strain (13,124, DSM 756) (NC_008261.1)	1	NEB HMW	STET (90%) + TE (10%) + lysozyme 10 mg/mL	1	Toxinotype A	–
		Reference strain (13,124, DSM 756) (NC_008261.1)	1	BT MasterPure	TE (100%) + lysozyme 10 mg/mL	1	Toxinotype A	–
		Reference strain (13,124, DSM 756) (NC_008261.1)	1	Promega HMW	TE (100%) + lysozyme 10 mg/mL	1	Toxinotype A	–
*C. perfringens*	CP2	Treated wastewater	1	NEB HMW	STET (90%) + TE (10%) + lysozyme 10 mg/mL	–	Toxinotype A	–
		Treated wastewater	1	BT MasterPure	TE (100%) + lysozyme 10 mg/mL	–	Toxinotype A	–
		Treated wastewater	1	Promega HMW	TE (100%) + lysozyme 10 mg/mL	–	Toxinotype A	–
*C. perfringens*	CP3	Bovine	2	BT MasterPure	TE (100%) + lysozyme 10 mg/mL	–	Toxinotype E	Plasmid carrying *iap*, *ibp*, *cpe*
*C. perfringens*	CP4	Human, stools	2	BT MasterPure	TE (100%) + lysozyme 10 mg/mL	–	Toxinotype G	Plasmid carrying *netB*

^*a*^ DNA extraction kits, lysis conditions, sequence types, toxin profiles, and the presence of plasmids are detailed for each strain. Plasmid presence was determined by PCR for *C. difficile* and inferred according to toxinotype for *C. perfringens*. DNA extraction kits: BT MasterPure, Biosearch Technologies MasterPure Complete DNA and RNA Purification Kit; NEB HMW, New England Biolabs Monarch High Molecular Weight DNA Extraction Kit for Tissue; Promega HMW, Promega High Molecular Weight DNA Extraction Kit.

All strains were characterized prior to sequencing according to previously described methods. Briefly, *C. difficile* and *C. perfringens* toxin profiles were determined by PCR following the protocols previously described (23, 24). Based on these results, the toxinotypes of *C. perfringens* CP3 and CP4 were identified according to a previous classification (7). These strains were expected to carry at least one plasmid. *C. difficile* CD3, isolated from a patient with *C. difficile* infection, was found to be resistant to metronidazole according to the CA-SFM guidelines (25). The presence of the plasmid pCD-METRO was determined by PCR as previously described (26).

### DNA extraction

Strains were stored at −80°C in cryotubes containing Brain Heart Infusion (BHI) (Oxoid; Thermo Fisher Scientific, Dardilly, France) supplemented with glycerol at a final concentration of 25%. *C. difficile* strains were restored from deep freezing on ChromID *C. difficile* agar (BioMérieux, Marcy-l’Etoile, France) and *C. perfringens* on CHROMagar *C. perfringens* (CHROMagar, Roubaix, France). Strains were incubated anaerobically in air-tight jars containing a sachet of Anaerogen anaerobic gas generator (Thermo Fisher Scientific) at 37°C for 24 h. A single colony was then enriched in 10 mL of BHI broth under the same incubation conditions. One milliliter of the overnight cultures was then centrifuged (5,000 × *g*, 10 min), and the resulting bacterial pellets were used for DNA extraction.

In experiment 1, the genomic DNA was extracted using three different kits (Fig. 1). An initial lysis step of 1 h at 37°C was performed using 10 mg/mL lysozyme (Merck, Saint-Quentin-Fallavier, France) as recommended for Gram-positive bacteria, using STET buffer (8% [wt/vol] sucrose, 50 mM Tris-HCl [pH 7.6] [Merck], 50 mM EDTA [Promega], and 5% Triton X-100 [Merck]) and/or TE buffer (10 mM Tris and 1 mM EDTA) (Table 1). DNA extraction was then performed according to the manufacturer’s instructions for the three different kits. In experiment 2, the DNA extraction was performed using only the BT MasterPure Kit under the same conditions as described above.

Purity and concentration of the extracted DNA were determined using a Nanodrop One spectrophotometer (Thermo Fisher Scientific, Saint-Herblain, France) and the Qubit Flex (Thermo Fisher Scientific) with the dsDNA High Sensitivity (HS) and Broad Range (BR) kits. Fragment lengths were determined using the 4200 TapeStation System (Agilent, Les Ulis, France) with the Genomic DNA ScreenTape kit.

### Library preparation and sequencing

Libraries were prepared using the Native Barcoding Kit 24 (v14, SQK-NBD114.24; ONT, Oxford, UK). DNA ligation was performed using the Next FFPE DNA Repair Mix and Ultra II End Repair/dA Tailing Module reagents (NEB). Libraries were then prepared using New England BioLabs reagents (Blunt/TA Ligase Master Mix, Next Quick Ligation Module) following the manufacturer’s instructions. DNA concentrations were measured at each step using the Qubit Flex with the HS Kit. Libraries were loaded onto R10 flow cells (FLO-MIN114, ONT), and sequencing was performed on a MinION Mk1B (ONT) device for 72 h. Data acquisition was carried out using MinKNOW software (v25.09.16), and basecalling was performed in real time using Dorado (v7.11.2) with the super-accurate basecalling 400 bps mode.

### Bioinformatic analyses

Raw and filtered data were analyzed using SeqKit (v2.9.0) (27). Raw data were filtered with Fitlong (v0.2.1) (28) with the following parameters: a minimum read length of 1,000 bp, a minimum read mean Phred quality score of 8, keeping 90% of best quality reads, and a target of 500 Mb of bases. Genomes were *de novo* assembled using Flye (v2.9.5) with the following parameters: nano-hq, one iteration, and 32 threads (29), and polished with Medaka (v2.0.1) using default parameters (github.com/nanoporetech/medaka). The benchmarking sets of universal single-copy orthologs score (BUSCO) was calculated using BUSCO (v6.0.0) (30). *C. difficile* strains were evaluated against the *Peptostreptococcaceae*_odb12 database and *C. perfringens* strains against the *Clostridiaceae*_odb12 database. STs were determined with *in silico* multilocus sequence typing (MLST) (31). Core genomes of *C. difficile* and *C. perfringens* strains were determined using previously existing schemes available on the core genome multilocus sequence typing (cgMLST) nomenclature server (cgmlst.org/ncs). We then used pyMLST (v2.1.6) to perform cgMLST (32). A minimum spanning tree was built with GrapeTree (33) using distances calculated from genes present in all strains (*n* = 1,957 genes for *C. difficile* and *n* = 1,399 genes for *C. perfringens*). Antimicrobial resistance genes and toxin genes were screened using ABRicate (v1.0.1) (34) with the following databases: VFDB (35), NCBI AMRFinderPlus (36), and MEGARES (37). For the detection of *netB*, BLAST (v2.16.0) was used, with the reference toxin sequence (EU143239.1).

### Statistical analyses

Differences in read length distribution and mean Phred score between extraction kits were assessed independently for each bacterial strain and filtering condition. All statistical analyses were performed in R (v4.4.1) using the rstatix package (v0.7.3). For each strain, Kruskal-Wallis tests were used to evaluate whether read length and Phred score distributions differed significantly across the three extraction kits. Due to highly unequal group sizes across conditions (number of reads (*n*) ranging from 11,156 to 573,354 per kit), for each pair of kits, 1,000 subsamples of size *n* = n_min (number of reads in the smallest group between the two compared kits) were drawn from the larger group, and pairwise comparisons were performed using Wilcoxon-Mann-Whitney tests and Benjamini-Hochberg correction. For all significant pairwise comparisons, *P* values were <0.001, and we then calculated effect sizes defined as *r* = |1–2*W* / (*n*₁ × *n*₂)|, where *W* is the result of the Wilcoxon-Mann-Whitney test, and *n*₁ = *n*₂ = n_min; *r* values are then averaged across the 1,000 iterations to obtain the final result. Effect size thresholds are defined as negligible (*r* < 0.10), small (0.10 ≤ *r* < 0.30), medium (0.30 ≤ *r* < 0.50), and large (*r* ≥ 0.50).

## RESULTS

Of the seven strains analyzed in this study, two (CD1 and CP1) were reference strains; the remaining five (CD2, CD3, CP2, CP3, and CP4) were clinical or environmental isolates. CD3, CP3, and CP4 were selected for their plasmid-encoded toxin or resistance gene content (Table 1).

### Experiment 1: comparative evaluation of DNA extraction kits

#### Genomic DNA quality and concentration

For the four strains CD1, CD2, CP1 and CP2, the highest DNA concentrations and quality were obtained using the BT MasterPure Kit, ranging from 55.8 to 100 ng/µL for both species (Table 2). The NEB HMW Kit produced the second-best yield for *C. difficile* (10.6–12.2 ng/μL), while the Promega HMW Kit produced the lowest yield (6.5–8.6 ng/μL). For *C. perfringens*, the Promega HMW Kit produced better results (85–90.2 ng/µL) than the NEB HMW Kit (63.5–51 ng/µL).

**TABLE 2:** DNA concentration and quality, and quality metrics of reads and assemblies for all strains sequenced in R1 and R2^*a*^

Species	Label	Extraction kit	DNA concentration (ng/μL)	260/280	260/230	DNA concentration (ng/μL) with the highest size (bp)	N50 (bp) before filtering	N50 (bp) after filtering	Average quality (Phred score) before filtering	Average quality (Phred score) after filtering	BUSCO score	Sequence type
*C. difficile*	CD1	NEB HMW	10.6	1.74	1.21	17.8–18,148; >60,000	20,204	24,146	18.65	19.2	C: 97.8% (S: 97.6%, D: 0.2%), F: 1.7%, M: 0.5%, n: 416	54
*C. difficile*		BT MasterPure	74.9	1.78	1.13	7.91–18,367; >60,000	22,497	41,364	18.99	22	C: 98.8% (S: 98.6%, D: 0.2%), F: 1.2%, M: 0.0%, n: 416	54
*C. difficile*		Promega HMW	6.5	2.03	3.8	3.57–26,696; >60,000	17,410	20,428	18.4	19.25	C: 98.8% (S: 97.8%, D: 1.0%), F: 1.2%, M: 0.0%, n: 416	54
*C. difficile*	CD2	NEB HMW	12.2	1.82	2.11	9.38–18,413; >60,000	12,560	16,008	18.47	19.04	C: 98.6% (S: 98.3%, D: 0.2%), F: 1.2%, M: 0.2%, n: 416	11
*C. difficile*		BT MasterPure	55.8	1.79	1.07	19.6–15,547; >60,000	20,608	36,177	19.1	21.87	C: 99.5% (S: 99.3%, D: 0.2%), F: 0.2%, M: 0.2%, n: 416	11
*C. difficile*		Promega HMW	8.6	2.11	2.9	14–20,161; >60,000	9,232	12,996	17.46	17.91	C: 98.8% (S: 98.6%, D: 0.2%), F: 0.7%, M: 0.5%, n: 416	11
*C. difficile*	CD3	BT MasterPure	84.6	–	–	–	4,739	10,818	21.35	21.73	C: 100.0% (S: 99.8%, D: 0.2%), F: 0.0%, M: 0.0%, n: 416	15
*C. perfringens*	CP1	NEB HMW	63.5	1.83	2.31	22.3–6,853; >60,000	11,047	45,266	18.37	22.93	C: 8.5% (S: 96.9%, D: 1.5%), F: 0.3%, M: 1.3%, n: 389	1
*C. perfringens*		BT MasterPure	100	1.82	1.85	259–11,435; >6,000	12,379	36,149	18.47	21.63	C: 98.7% (S: 97.2%, D: 1.5%), F: 0.3%, M: 1.0%, n: 389	1
*C. perfringens*		Promega HMW	85	2.13	2.38	22.9–7,460; >60,000	27,820	42,511	18.98	22.28	C: 98.7% (S: 97.2%, D: 1.5%), F: 0.3%, M: 1.0%, n: 389	1
*C. perfringens*	CP2	NEB HMW	51	1.83	1.89	45.7–10.193; >60,000	9,546	28,350	18.64	21.81	C: 98.2% (S: 96.9%, D: 1.3%), F: 0.5%, M: 1.3%, n: 389	1246
*C. perfringens*		BT MasterPure	100	1.82	1.47	180–8,772; >60,000	10,589	37,305	18.89	22.08	C: 98.2% (S: 96.9%, D: 1.3%), F: 0.5%, M: 1.3%, n: 389	1246
*C. perfringens*		Promega HMW	90.2	2.14	2.25	6.2–10,196; >60,000	29,780	43,671	18.58	22.38	C: 98.5% (S: 97.2%, D: 1.3%), F: 0.3%, M: 1.3%, n: 389	1246
*C. perfringens*	CP3	BT MasterPure	353	–	–	–	11,560	28,018	22.21	24.15	C: 99.0% (S: 97.9%, D: 1.0%), F: 0.0%, M: 1.0%, n: 389	141
*C. perfringens*	CP4	BT MasterPure	602	–	–	–	16,845	20,197	22.28	22.72	C: 98.5% (S: 96.4%, D: 2.1%), F: 0.5%, M: 1.0%, n: 389	122

^*a*^ DNA concentration and purity were measured using the Qubit (High Sensitivity and Broad Range kits) and Nanodrop (260/280 and 260/230 ratios), respectively. Sequencing read quality (average Phred score) and N50 values were calculated using SeqKit, and genome completeness was assessed using BUSCO. C, complete; D, duplicated; F, fragmented; M, missing; S, single.

The TapeStation analysis revealed that the three kits produced fragments of a large size. The Promega HMW Kit produced slightly larger fragments for *C. difficile*, with dominant fragment sizes from around 26 to >60 kb and from 20 to >60 kb. In contrast, the NEB HMW Kit and the BT MasterPure Kit produced DNA with slightly shorter fragments, ranging from 15 and 18 kb to >60 kb, respectively (Table 2). For *C. perfringens*, the BT MasterPure Kit produced the largest fragments for CP1 (approximately from 11 to >60 kb) and the smallest for CP2 (approximately from 8 to >60 kb). The NEB HMW Kit and the Promega HMW Kit produced similar results, with fragment sizes ranging from ~7 to >60 kb for CP1 and from ~10 to >60 kb for CP2 (Table 2).

#### Sequencing performances

**Fig 2:**
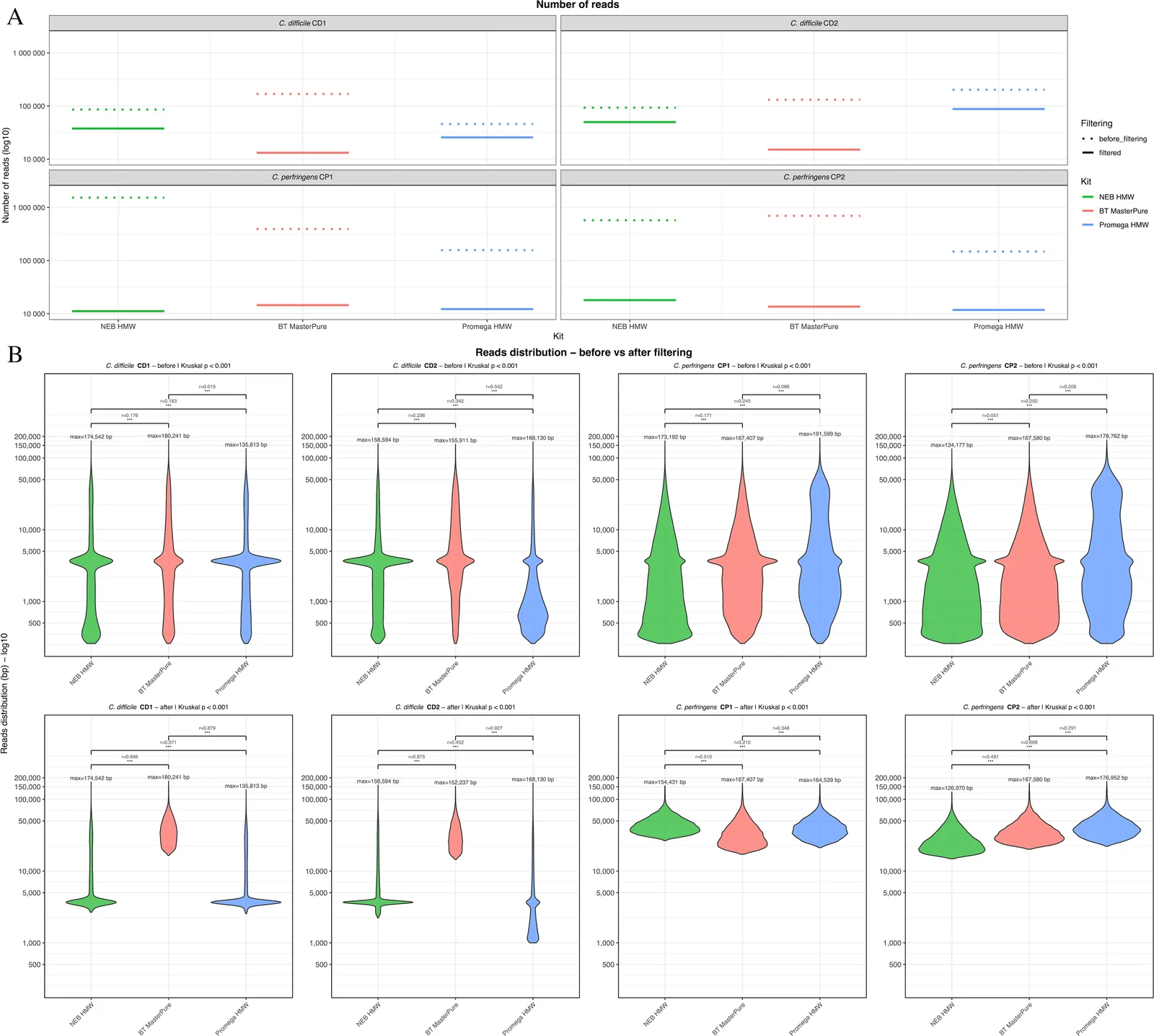
Read quality obtained before and after filtering for the four different strains sequenced in experiment 1. Number of reads (**A**) and read length distribution (**B**) have been determined for the two *Clostridioides difficile* (CD1 and CD2) and the two *Clostridium perfringens* (CP1 and CP2) and for the three different kits. In panel **B**, global differences across kits were assessed using Kruskal-Wallis tests (*P* value indicated in each panel title). Pairwise comparisons were performed using Wilcoxon-Mann-Whitney tests on 1,000 balanced random subsamples; effect sizes (*R*) and significance levels (***, *P* < 0.001) are displayed above each comparison bracket. The maximum read length observed for each kit is indicated above each violin. Violin shapes were trimmed to the observed data range. BT MasterPure, Biosearch Technologies MasterPure Complete DNA and RNA Purification Kit; NEB HMW, New England Biolabs Monarch High Molecular Weight DNA Extraction Kit for Tissue; Promega HMW, Promega Wizard High Molecular Weight DNA Extraction Kit.

For *C. difficile*, the number of reads before filtering obtained ranged from 45,729 to 202,743, depending on the strain and extraction kit. The highest number of reads was obtained with the Promega HMW Kit for CD2 (202,743) and with the BT MasterPure Kit for CD1 (168,876) (Fig. 2A). The number of reads for *C. perfringens* was much higher than that for *C. difficile*, ranging from 147,766 to 1,525,547. The maximum number of reads was obtained for CP1 using the NEB HMW Kit, while CP2 reached 693,477 using the BT MasterPure Kit (Fig. 2A).

Before filtering, *C. difficile* read length distributions differed significantly across extraction kits for both CD1 and CD2. For CD1, the Promega HMW Kit produced longer reads than both the BT MasterPure Kit and the NEB HMW Kit but with small (*r* = 0.176) and negligible (*r* = 0.019) effect sizes, respectively. For CD2, differences were more pronounced: the BT MasterPure Kit produced significantly longer reads than the Promega HMW Kit with a large effect size (*r* = 0.542), as did the NEB HMW Kit with a small effect size (*r* = 0.236) (Fig. 2B). Kit-related differences were amplified after quality filtering for both strains. The BT MasterPure Kit consistently produced the longest reads, showing large effect sizes compared to the NEB HMW Kit (CD1: *r* = 0.846, CD2: *r* = 0.875) and the Promega HMW Kit (CD1: *r* = 0.879, CD2: *r* = 0.927) (Fig. 2B). Maximum read lengths were comparable across kits, ranging from 135 to 180 kb. After filtering, the longest read was obtained with the BT MasterPure Kit for CD1 (180,241 bp) and with the Promega HMW Kit for CD2 (168,130 bp) (Fig. 2B).

Before filtering, *C. perfringens* CP1 and CP2 showed statistically significant kit effects on read length. For CP1, the BT MasterPure Kit produced longer reads than the NEB HMW Kit with a small effect size (*r* = 0.171). The Promega HMW Kit outperformed both the NEB HMW Kit and the BT MasterPure Kit with small (*r* = 0.245) and negligible (*r* = 0.086) effect sizes, respectively. For CP2, the Promega HMW Kit produced slightly longer reads than both the NEB HMW Kit (*r* = 0.250) and the BT MasterPure Kit (*r* = 0.209) (Fig. 2B). Kit effects were substantially amplified after filtering for both strains. For CP1, the NEB HMW Kit produced longer reads than the BT MasterPure Kit with a large effect size (*r* = 0.510) and also outperformed the Promega HMW Kit with a small effect size (*r* = 0.210). For CP2, the Promega HMW Kit produced the longest reads overall, surpassing both the NEB HMW Kit with a large effect size (*r* = 0.668) and the BT MasterPure Kit with a small effect size (*r* = 0.291), while the BT MasterPure Kit yielded longer reads than the NEB HMW Kit with a large effect size (*r* = 0.491). After filtering, the longest read was obtained with the BT MasterPure Kit for CP1 (167,407 bp) and with the Promega HMW Kit for CP2 (176,952 bp) (Fig. 2B).

The DNA sequencing read quality and contiguity were evaluated for each strain. The read-length N50 values (defined as the minimum read length such that 50% of the total sequenced bases are contained in reads of that length or longer) prior to filtering varied across species and extraction methods, ranging from 9,232 to 22,497 bp for *C. difficile* and from 9,546 to 29,780 bp for *C. perfringens*. After filtering, the read-length N50 values increased substantially, with the highest read-length N50 reaching 45,266 bp for CP1 extracted with the NEB HMW Kit, 43,671 bp for CP2 with Promega HMW Kit, and 41,364 and 36,177 for CD1 and CD2 with BT MasterPure Kit, respectively.

Phred scores, defined as the mean per-base quality score within each read and related to the base-calling error probability, were determined. A Phred score of 20 corresponds to a predicted error rate of 1%, indicating a 99% chance of accuracy. Before filtering, *C. difficile* mean Phred score distributions differed significantly across extraction kits for CD1 and CD2. Average Phred scores ranged from 17.46 to 19.1 across conditions before filtering (Table 2).

For CD1, differences were small to negligible, with the BT MasterPure Kit showing slightly higher Phred scores than the NEB HMW Kit (*r* = 0.138), while all other comparisons yielded negligible effect sizes (*r* ≤ 0.075) (Fig. S1). For CD2, differences were more pronounced, with the BT MasterPure Kit outperforming both the NEB HMW Kit and the Promega HMW Kit with small (*r* = 0.177) and medium (*r* = 0.424) effect sizes, respectively (Fig. S1). The highest Phred scores before filtering were obtained using the BT MasterPure Kit for CD1 (18.99) and CD2 (19.1) (Table 2).

After filtering, average Phred scores ranged from 17.91 to 22.00 (Table 2), and kit-related differences were amplified. For CD1, the BT MasterPure Kit outperformed both the NEB HMW Kit and the Promega HMW Kit with medium effect sizes (*r* = 0.431 and *r* = 0.481, respectively) (Fig. S1). For CD2, the BT MasterPure Kit systematically produced the highest-quality reads, with a large effect size compared to the Promega HMW Kit (*r* = 0.673) and a medium effect size compared to the NEB HMW Kit (*r* = 0.477) (Fig. S1). The highest Phred scores after filtering were obtained using the BT MasterPure Kit for both CD1 (22.00) and CD2 (21.87) (Table 2).

For *C. perfringens*, average Phred scores ranged from 18.37 to 18.98 before filtering (Table 2). For CP1, the Promega HMW Kit produced slightly higher quality reads than both the BT MasterPure Kit (*r* = 0.027) and the NEB HMW Kit (*r* = 0.141). For CP2, all effect sizes were negligible (*r* ≤ 0.067) (Fig. S1). The highest Phred scores before filtering were obtained using the Promega HMW Kit for CP1 (18.98) and the BT MasterPure Kit for CP2 (18.89) (Table 2).

After filtering, average Phred scores ranged from 21.63 to 22.93 (Table 2). For CP1, the NEB HMW Kit produced slightly higher quality reads than both the BT MasterPure Kit (*r* = 0.190) and Promega HMW Kit (*r* = 0.122). For CP2, the Promega HMW Kit and the BT MasterPure Kit were equivalent with a negligible effect size and slightly outperformed the NEB HMW Kit (*r* < 0.15) (Fig. S1). The highest Phred scores after filtering were obtained using the NEB HMW Kit for CP1 (22.93) and the Promega HMW Kit for CP2 (22.38) (Table 2).

#### Evaluation of assembled genomes

**Fig 3:**
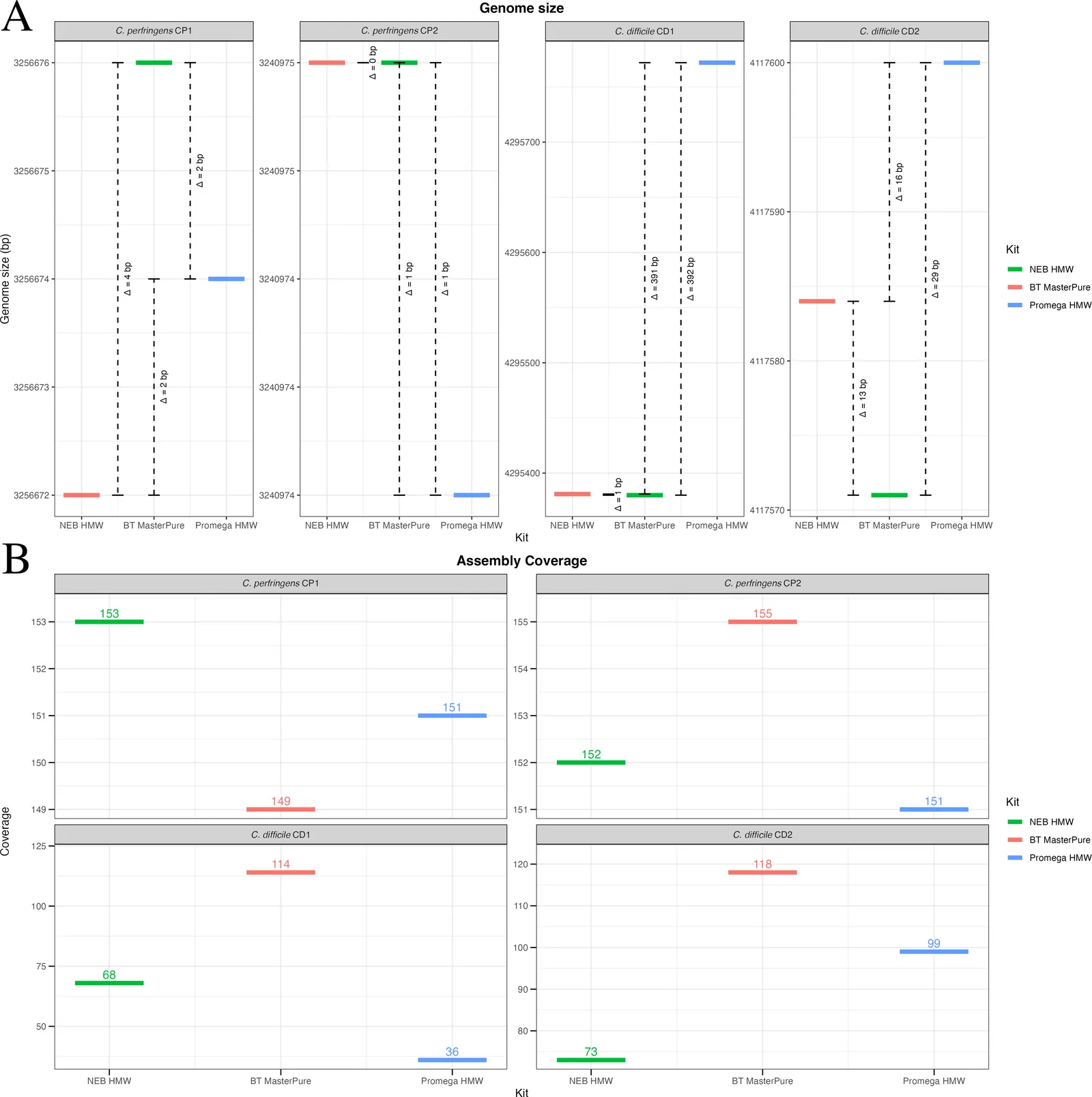
Genome size and coverage obtained after assembly for the four different strains sequenced in experiment 1. Differences in size between assembled genomes (**A**) and assembly coverages (**B**) obtained for each strain, depending on the kit used for DNA extraction. Δ indicates the difference in size between genomes (bp). BT MasterPure, Biosearch Technologies MasterPure Complete DNA and RNA Purification Kit; NEB HMW, New England Biolabs Monarch High Molecular Weight DNA Extraction Kit for Tissue; Promega HMW, Promega Wizard High Molecular Weight DNA Extraction Kit.

All genomes were successfully assembled and circularized, regardless of the DNA extraction kit used. Minor differences in assembly length were observed between kits, ranging from 0 to 4 bp for *C. perfringens* (CP1 and CP2) (Fig. 3A). For *C. difficile* CD1, the genomes obtained with the BT MasterPure Kit and the NEB HMW Kit differed by only 1 bp in length. By contrast, the genomes obtained using the Promega HMW Kit were 391 and 392 bp longer than those obtained with the NEB HMW Kit and the MasterPure Kit, respectively. CD2 showed only minor differences in genome length, ranging from 13 to 29 bp between kits (Fig. 3A).

All *C. perfringens* strains reached a coverage of >149×. In contrast, substantial differences in sequencing coverage were observed for CD1 and CD2, depending on the DNA extraction kit used. The BT MasterPure Kit produced the highest coverage for both strains (114× for CD1 and 118× for CD2). Assembly coverages using the NEB HMW Kit were 68× for CD1 and 73× for CD2, and 36× for CD1 and 99× for CD2 using the Promega HMW Kit (Fig. 3B).

Genome completeness was evaluated using BUSCO (Table 2). A total of 414 genes contained in the *Peptostreptococcaceae*_odb12 database for *C. difficile* and 389 genes in the *Clostridiaceae*_odb12 database for *C. perfringens* were used to assess genome quality and completeness. No matter which kit was used, BUSCO identified >97.8% of the genes of *C. difficile* and *C. perfringens* (Table 2). BUSCO analysis confirmed a high level of genome completeness for all *C. difficile* assemblies, with the best results obtained using the BT MasterPure Kit. This kit identified an average of 413 ± 2.12 complete genes, corresponding to 99.16% ± 0.51% completeness, compared with 411 ± 0 genes (98.8% ± 0%) for the Promega HMW Kit and 409 ± 2.12 genes (98.2% ± 0.51%) for the NEB HMW Kit. Using the BT MasterPure Kit, we obtained the lowest proportion of fragmented genes, with an average of 3 ± 0.71 genes (0.72% ± 0.17%), whereas the Promega HMW Kit and the NEB HMW Kit showed higher values of 6 ± 1.41 genes (1.44% ± 0.34%) and 6 ± 2.83 genes (0.72% ± 0.68%), respectively. Similarly, the number of missing BUSCO genes remained low with all kits but was the lowest using the BT MasterPure Kit, with 1 ± 0.71 genes (0.12% ± 0.17%), compared with 1 ± 1.41 genes (0.24% ± 0.34%) with the Promega HMW Kit and 2 ± 0.71 genes (0.36% ± 0.17%) with the NEB HMW Kit (Table 2). BUSCO analysis also confirmed a high level of genome completeness for all *C. perfringens* assemblies, with comparable results across the three extraction kits (Table 2). The Promega HMW Kit identified an average of 384 ± 0.71 complete genes, corresponding to 98.59% ± 0.17% completeness, while the NEB HMW Kit and the BT MasterPure Kit identified 383 ± 0.71 genes (98.33% ± 0.17%) and 383 ± 1.41 genes (98.46% ± 0.34%), respectively. The proportion of fragmented genes was low for all kits, with an average of 1 ± 0 gene (0.26% ± 0%) using the Promega HMW Kit and 2 ± 0.71 genes (0.39% ± 0.18%) for both the NEB HMW Kit and BT MasterPure Kit. Similarly, the number of missing BUSCO genes remained low no matter the kit used, with 5 ± 0.71 genes (1.16% ± 0.18%) for the Promega HMW Kit, 5 ± 0.00 genes (1.29% ± 0.00%) for the NEB HMW Kit, and 5 ± 0.71 genes (1.16% ± 0.18%) for the BT MasterPure Kit (Table 2).

#### Genomic characterization

**Fig 4:**
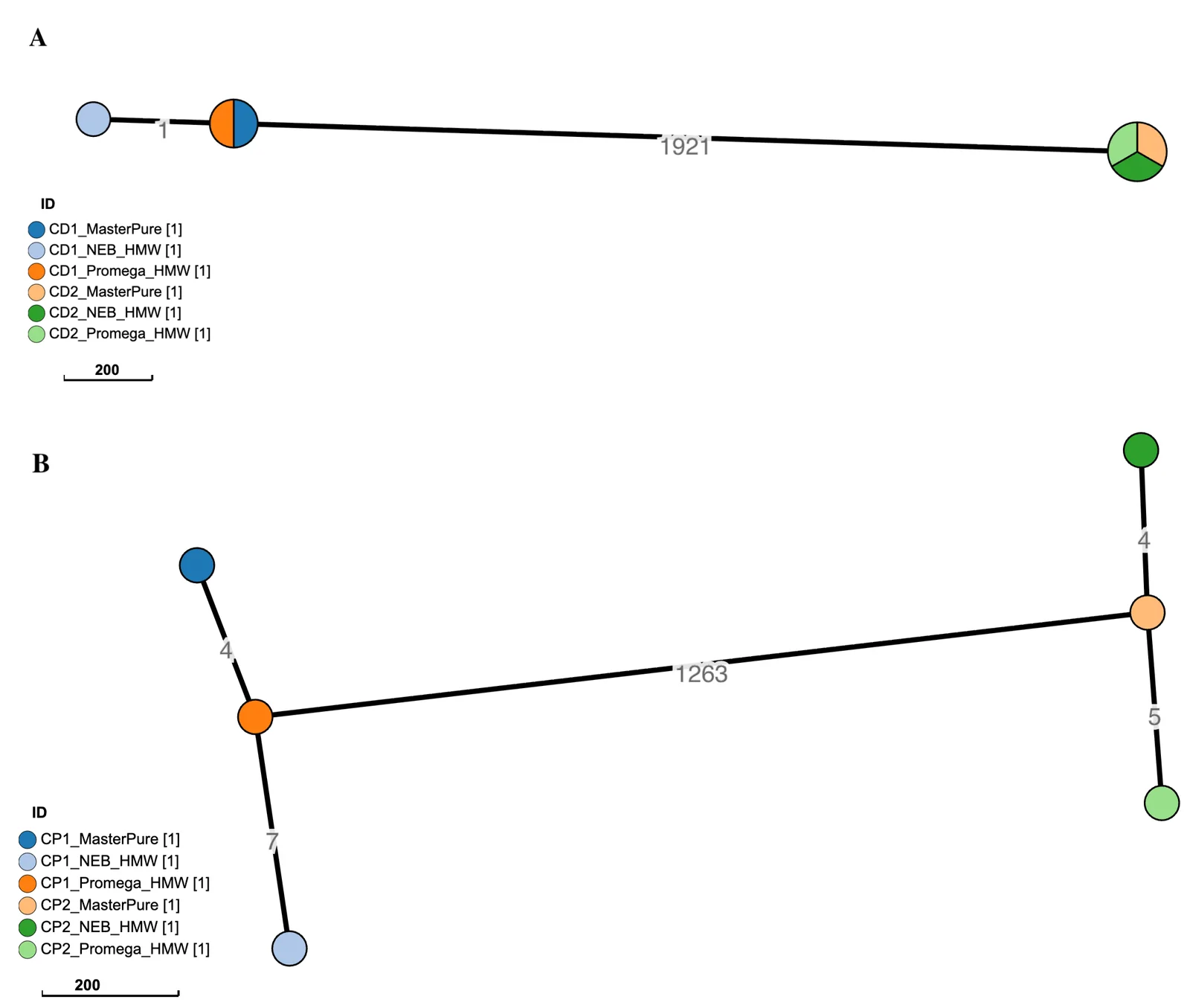
Minimum spanning trees (MSTs) based on the core genome multilocus sequence typing (cgMLST) analyses obtained with the three different DNA extraction kits in experiment 1. MST based on the cgMLST analysis of two *C. difficile* strains (**A**) and two *C. perfringens* strains (**B**) sequenced after DNA extraction with the three different kits. Numbers on branches indicate the number of alleles by which the strains differ. BT MasterPure, Biosearch Technologies MasterPure Complete DNA and RNA Purification Kit; NEB HMW, New England Biolabs Monarch High Molecular Weight DNA Extraction Kit for Tissue; Promega HMW, Promega Wizard High Molecular Weight DNA Extraction Kit.

To verify the quality of the sequencing data for the strains sequenced, we first checked that the STs had been assigned correctly for the two reference strains and characterized the STs of the other strains. Second, we compared the core genomes of each strain and searched for ARGs and toxin-encoding genes. Regardless of the kit used, assigned STs were identical to the original STs for the two reference strains (Table 1). The STs of the other strains are shown in Table 2. For strains CD2 and CP2, STs were consistent across the three extraction kits. On the 2,147 genes analyzed in the core genome analysis of *C. difficile*, CD1 extracted with the BT MasterPure Kit and the Promega HMW Kit showed identical core genomes, whereas 1 gene was different using the NEB HMW Kit (Fig. 4A). For CD2, core genomes were identical, independently of the kit used (Fig. 4A). Note that CD1 and CD2 clusters were separated by a distance of 1,911 alleles (Fig. 4A). We found more differences in the core genomes of *C. perfringens*. On the 1,431 genes analyzed, strains differed by four to seven alleles, depending on the kit used (Fig. 4B). CP1 and CP2 clusters were separated by a distance of 1,208 alleles (Fig. 4B). All ARGs and toxin-encoding genes were detected with sequence identities greater than 95% (Table S1). Importantly, no differences in gene presence, percentage identity, or coverage were observed between DNA extraction kits for a given strain.

### Experiment 2: plasmid recovery efficiency

#### DNA quality, sequencing performances, and assembly

Based on DNA and read quality, as well as kit ease of use, the BT MasterPure Kit was selected for DNA extraction of *C. difficile* CD3 and *C. perfringens* CP3 and CP4 in order to assess its ability to recover plasmids and confirm the abovementioned performances on full genome recovery when followed by Oxford Nanopore sequencing. DNA concentrations were 84.6, 353, and 602 ng/μL for CD3, CP3, and CP4, respectively (Table 2).

DNA sequencing of CD3, CP3, and CP4 resulted in 484,579, 448,347, and 80,983 reads, respectively, before filtering, and 67,442, 19,724, and 37,445 reads, respectively, after filtering. The average read lengths were 1,560 bp (CD3), 2,432 bp (CP3), and 7,040 bp (CP4) before filtering, and 7,413 bp (CD3), 23,350 bp (CP3), and 13,353 bp (CP4) after filtering. No difference in maximum read lengths was observed before and after filtering, with 143,913 bp for CD3, 156,708 bp for CP3, and 147,663 bp for CP4 (Table 2). Prior to read filtering, the read-length N50 value was highest for CP4 at 16,845 bp, followed by CP3 at 11,560 bp, and CD3 at 4,739 bp. Following filtering, the read-length N50 value increased for all samples, reaching 28,018 bp for CP3, 20,197 bp for CP4, and 10,818 bp for CD3. The average Phred scores also improved after filtering. Before filtering, the scores were 21.35 for CD3, 22.21 for CP3, and 22.28 for CP4. Following filtering, these scores increased to 21.73 for CD3, 24.15 for CP3, and 22.72 for CP4 (Table 2).

Genomes of CD3, CP3, and CP4 were all successfully assembled and circularized, with genome sizes and assembly coverage of 4,258,023 bp and 110×, 3,462,662 bp and 139×, and 3,298,770 bp and 141×, respectively. We also identified three circularized plasmids in strain CD3 (33,914 bp, 221× coverage; 7,991 bp, 324× coverage; and 7,056 bp, 1,203× coverage) and one circularized plasmid in the strain CP3 (67,478 bp, 125× coverage). For CP4, we identified two uncircularized plasmids (82,396 bp at 189× and 50,990 bp at 86× coverage).

The BUSCO analysis of the assemblies generated in experiment 2 also demonstrated excellent genome completeness. For CP3, 385 complete BUSCO genes were identified out of 389 (99.0%), with no fragmented genes and only 4 missing genes (1.0%). CP4 also showed high completeness, with 383 complete BUSCO genes (98.5%), with two fragmented BUSCO genes (0.5%) and 4 missing genes (1.0%). The CD3 genome reached full completeness, with 416 complete BUSCO genes out of 416 (100.0%), and so no fragmented or missing BUSCO genes (Table 2).

#### Genomic characterization

STs, ARGs, and toxin-encoding genes were characterized for CD3, CP3, and CP4. STs are presented in Table 2. All ARGs and toxin-encoding genes expected were detected with sequence identities greater than 95% (Table S1).

Antibiotic susceptibility to metronidazole and PCR detection of pCD-METRO in CD3, as well as the toxinotypes identified for strains CP3 and CP4, indicate that these strains carry plasmids. The putative plasmids detected in these strains were subsequently analyzed using BLAST. The three plasmids identified in CD3 corresponded to previously characterized *C. difficile* plasmids. In particular, the 7,056 bp plasmid was confirmed as pCD-METRO, with 100% sequence identity and 100% coverage. No genes encoding toxins were found on the chromosome or plasmids for CD3. For CP3, the toxin genes *cpe*, *iap*, and *ibp*, which are known to be plasmid-encoded, were detected on a 67,478 bp plasmid. This plasmid was identified as pCPPB-1 (99.9% identity, 100% coverage). For CP4, the tetracycline resistance genes *tetB(P*) and *tetA(P)* were detected on a 50,990 bp putative plasmid. BLAST analysis showed that this plasmid was closely related to pDel1_2, with 98.04% sequence identity and 86% coverage. In addition, the necrotic enteritis B toxin gene (*netB*) was carried on an 82,396 bp putative plasmid, which was identified as pCP280-82k (100% identity, 97% coverage).

## DISCUSSION

The use of Oxford Nanopore sequencing for bacterial whole-genome sequencing has become a standard for generating long reads, allowing complete genome assembly and the precise identification of the genetic structures of ARGs and mobile genetic elements such as plasmids (21). *C. difficile* and *C. perfringens* are known to harbor a large diversity of MGEs, particularly plasmids, which often carry virulence factors and ARGs, making long-read sequencing especially valuable for accurately resolving these elements (7, 13, 14). To date, *C. difficile* and *C. perfringens* genomes have mainly been sequenced using short-read technologies or hybrid approaches combining short- and long-read sequencing (15, 17, 38, 39). The Gram-positive cell wall structure and spore-forming ability of these species can make DNA extraction challenging, often resulting in fragmented or low-quality DNA, particularly when bead-beating extraction methods are used (40). However, long-read sequencing requires high-quality and minimally fragmented DNA.

The aim of this study was to identify the most effective DNA extraction method for long-read sequencing of *C. difficile* and *C. perfringens*. Overall, the three DNA extraction kits evaluated in this study enabled complete genome assembly and reliable identification of ARGs, toxin-encoding genes, and STs for both *C. difficile* and *C. perfringens*. However, differences were observed in sequencing performances, particularly for *C. difficile*. The BT MasterPure Kit provided a slightly higher DNA yield and longer DNA fragments, resulting in better average read length, higher N50 values, and better Phred scores. Previous studies showed that the BT MasterPure Kit produced better results for the sequencing of *Escherichia coli* and *Bacillus anthracis*, with Nanopore sequencing than other commercial kits (41, 42). After assembly, this kit also consistently achieved genome coverages above 100× for both species, whereas the two other kits produced lower coverages for *C. difficile*. The high coverage obtained with the BT MasterPure Kit was correlated with higher BUSCO completeness scores, indicating a low assembly error rate and a high genome quality. While the three DNA extraction kits provided similar results for *C. perfringens*, with comparable genome assembly quality metrics and consistent downstream analysis outcomes, this was not the case for *C. difficile*. DNA yields were lower, and the extracted DNA appeared more fragmented for *C. difficile* than for *C. perfringens,* with variations between kits. Several hypotheses can be proposed to explain these differences. Although the initial culture volumes and growth conditions were standardized for both species, *C. perfringens* is relatively easy to cultivate and has a short generation time, allowing for the production of substantial biomass, resulting in a greater quantity and higher quality of DNA (43). In contrast, *C. difficile* has more specific growth requirements, which may have resulted in lower biomass production compared to *C. perfringens*. Another very important factor that may have contributed to these differences is the ratio of vegetative cells to spores in the initial culture used for DNA extraction, which was not assessed in our study. Spores are indeed known to be more difficult to lyse to extract DNA from than vegetative cells (44). Overnight *C. difficile* cultures are expected to contain a mixture of spores and vegetative cells (22, 44). Furthermore, the induction of *C. perfringens* sporulation under *in vitro* conditions can be challenging and varies significantly among strains and culture conditions (45). The presence of *C. perfringens* spores under the conditions used here is highly unlikely, which could therefore contribute to the differences observed between the two bacteria. In this context, the results show that the BT MasterPure Kit performed better than other kits, at least for *C. difficile*.

The minimum coverage obtained for *C. difficile* in this study was 36× for CD1 using the Promega HMW Kit. This coverage was sufficient to achieve complete genome circularization, for accurate ST determination and identification of AMR and toxin-coding genes, and to obtain a cgMLST profile similar to those obtained with other kits. This suggests that 36× coverage is sufficient for bacterial typing and epidemiological investigations. Nevertheless, further studies are needed to establish the minimum coverage necessary for precise bacterial typing of *C. difficile* and *C. perfringens* using Oxford Nanopore sequencing. Previous studies on other bacterial species have reported the need for significantly higher coverage for long-read-only assemblies, recommending depths of 75× or 100× to ensure optimal assembly quality (with Native Barcoding Kit 24 v14 and R10 flow cells) (46, 47). Here, with the same generation of flow cells but with the Dorado Super Accurate Model, the sequencing accuracy has improved compared to older technologies. This explains why a coverage of 36× was sufficient for accurate bacterial typing. A similar coverage (35×) was recently shown to be sufficient to provide cgMLST error rates of less than 0.5% for different bacterial species (*Klebsiella pneumoniae*, *Bordetella pertussis*, and *Corynebacterium diphtheriae*) (48).

In experiment 2, the BT MasterPure Kit confirmed its performances for genome assembly of one *C. difficile* (CD3) and two *C. perfringens* (CP3 and CP4). Moreover, it enabled the complete recircularization of the clinically relevant pCD-METRO plasmid (7,056 bp) of CD3, which confers resistance to metronidazole (14). In *C. perfringens* CP3, this approach also allowed the identification and recircularization of the 67,478 bp pCPPB-1 plasmid encoding the toxin genes *cpe*, *iap*, and *ibp* (49). In addition, two other putative plasmids were identified in *C. perfringens* CP4, carrying genes associated with tetracycline resistance [*tetB(P)* and *tetA(P)*] and the necrotic enteritis B toxin gene (*netB*). These results demonstrate the suitability of this method for the characterization of ARGs and toxin-encoding genes and of their genetic environments. It should be noted that, with the exception of the CD3 pCD-METRO plasmid, whose presence was independently confirmed by PCR prior to sequencing (data not shown), plasmid characterization in *C. perfringens* strains relied on sequencing-based evidence. The presence of plasmids in CP3 and CP4 is nonetheless independently supported by their toxinotype, as the corresponding toxin genes are known to be exclusively plasmid-encoded in this species (7). Confidence in full structural resolution is highest for the circularized CP3 plasmid (pCPPB-1, 99.9% identity) and comparatively lower for the two non-circularized CP4 plasmids. Their non-circularization most likely reflects the presence of repetitive elements at the contig ends and can prevent assembly algorithms from unambiguously resolving the circular junction despite adequate sequencing coverage.

Current challenges in the genomic surveillance of *C. difficile* and *C. perfringens* include the detection of plasmid-encoded resistance determinants, the characterization of toxin-encoding mobile elements, and the tracking of emerging hypervirulent clones. The optimized DNA extraction and long-read sequencing workflow described here provides a methodological basis for addressing these challenges, with the potential to support future real-time genomic surveillance of these clinically important pathogens. Previous studies on various bacterial pathogens showed that Oxford Nanopore sequencing enables high-resolution typing, pathogen surveillance, and outbreak detection linked to several bacterial species (50–52). We also compared the performance of the three DNA extraction kits in terms of processing time and technical complexity. The Promega HMW Kit had the fastest processing time and was relatively easy to implement. The BT MasterPure Kit required slightly more hands-on time but was still easy to use and the most cost-effective option per sample. By contrast, the NEB HMW Kit was the most time-consuming and technically demanding, with the highest cost. Overall, the BT MasterPure Kit offered the best balance of efficiency, simplicity and affordability.

The main limitation of this study was the number of strains that were sequenced; the inclusion of greater genetic and plasmid diversity would have facilitated the generalization of the results. In addition, this study did not include technical or biological replicates for each strain/kit combination, so the observed differences between kits cannot be fully disentangled from extraction-to-extraction or run-to-run variability. Extending this comparison to a larger and more clinically and environmentally diverse strain collection, including replicate extractions and sequencing runs, represents an important direction for future work. In addition, this study was conducted in a single laboratory using a single set of equipment and operators. Inter-laboratory variability, related to differences in equipment, reagent lots, or operator handling, could influence DNA yield and sequencing performance and was not assessed here. Multicentric studies would be needed to definitively confirm the generalizability of the relative kit performance reported in this study. Nevertheless, the results for the 15 strains sequenced were robust, with complete chromosome assembly and comprehensive strain characterization.

In conclusion, to overcome the challenges of extracting high-molecular-weight DNA from Gram-positive, spore-forming bacteria such as *C. difficile* and *C. perfringens* for Oxford Nanopore sequencing, we compared three commercially available extraction kits. All three kits produced DNA suitable for long-read sequencing. However, the Biosearch Technologies MasterPure Complete DNA and RNA Purification Kit (BT MasterPure Kit), when used in combination with a preliminary lysozyme treatment, proved to be the most effective option overall. It consistently produced high-quality and high-integrity DNA, enabling the generation of sequencing data that allowed for complete chromosome circularization, >100× sequencing depth, and recovery of complete plasmid sequences. Importantly, it also enabled the reliable identification of STs, toxin-encoding genes, and ARGs, ensuring the accurate characterization of clinically relevant genomic features. Taken together, our findings provide a practical, cost-effective framework for optimizing the long-read whole-genome sequencing of clinically important spore-forming pathogens.

## Supplementary Material

Reviewer comments

## Data Availability

Sequencing data generated in this study for the 15 strains of *Clostridioides difficile* and *Clostridium perfringens* are available in the NCBI database under BioProject accession number PRJNA1441429 (Evaluation of DNA extraction kits for Nanopore sequencing of *C. difficile* and *C. perfringens*).

## SUPPLEMENTAL MATERIAL

Fig. S1 spectrum.02407-26-s0001.tif Phred scores.

Supplemental legends spectrum.02407-26-s0002.docx Legends for Table S1 and Fig. S1.

Table S1 spectrum.02407-26-s0003.xlsx Antimicrobial resistance genes and toxin genes identified in genomes of *C. difficile* (CD1 and CD2) and C. perfringens (CP1 and CP2) strains.

OPEN PEER REVIEW reviewer-comments.pdf An accounting of the reviewer comments and feedback.

ASM does not own the copyrights to Supplemental Material that may be linked to, or accessed through, an article. The authors have granted ASM a non-exclusive, world-wide license to publish the Supplemental Material files. Please contact the corresponding author directly for reuse.
