# Supplementary material for: Comparative evaluation of three high-molecular-weight DNA extraction kits for Oxford Nanopore sequencing of Clostridioides difficile and Clostridium perfringens
Source: Microbiol Spectr. 2026 Sep 11;14(10):e02407-26. doi: 10.1128/spectrum.02407-26 (PMC13688846; doi:10.1128/spectrum.02407-26)
Supplement: Supplementary file 1 [file reviewer-comments.pdf]

ASM | Microbiology Spectrum

Comparative evaluation of three high-molecular-weight DNA extraction methods for optimal Oxford Nanopore long-read sequencing of the Gram-positive spore-forming Clostridioides difficile and Clostridium perfringens

Alexandre Mrozinski, Daniel Martak, Guillaume Reboul, Muriel Ehmig, Laure Diancourt, Frederic Barbut, Caroline Le Marechal, and Edward Topp

Corresponding Author(s): Edward Topp, Institut National de Recherche pour l'Agriculture l'Alimentation et l'Environnement Centre Bourgogne-Franche-Comte

Review Timeline:

Submission Date:	July 13, 2026
Editorial Decision:	July 18, 2026
Revision Received:	July 21, 2026
Accepted:	July 31, 2026

Editor: Erik Hom

Reviewer(s): The reviewers have opted to remain anonymous.

Transaction Report:

(Note: With the exception of the correction of typographical or spelling errors that could be a source of ambiguity, letters and reports are not edited. The original formatting of letters and referee reports may not be reflected in this compilation.)

DOI: https://doi.org/10.1128/spectrum.02407-26

Re: Spectrum02407-26 (Comparative evaluation of three high-molecular-weight DNA extraction methods for optimal Oxford Nanopore long-read sequencing of the Gram-positive spore-forming Clostridioides difficile and Clostridium perfringens)

Dear Dr. Edward Topp:

Thank you for the privilege of reviewing your work. I am pleased to inform you that your manuscript has been editorially accepted for publication. However, there are a few additional questions in the submission form that need to be answered before your manuscript is officially accepted. Once these are completed, please return your submission so that I can move your paper forward to acceptance.

Please return the manuscript within 60 days; if you cannot complete the submission questions within this time period, please contact me.

Thank you for submitting your paper to Spectrum.

Sincerely,
Erik Hom
Editor
Microbiology Spectrum

Response to Reviewers

Comparative evaluation of three high-molecular-weight DNA extraction kits for Oxford Nanopore sequencing of *Clostridioides difficile* and *Clostridium perfringens*

Alexandre Mrozinski*, Daniel Martak*, Guillaume Reboul, Muriel Ehmig, Laure Diancourt, Frédéric Barbut, Caroline Le Maréchal, Edward Topp

We thank all three reviewers for their careful reading of the manuscript and their constructive comments. We have addressed each point below. Reviewer comments are shown in italics, our responses follow each comment, and are indicated in bold.

REVIEWER #1

The study addresses an important methodological question regarding DNA extraction for long-read sequencing of Clostridioides difficile and Clostridium perfringens, and provides useful comparative data across three commercially available kits. However, the study is limited by the small number of strains analyzed (n = 4). This restricts the ability to draw robust conclusions regarding the relative performance of the extraction methods and may not adequately capture the biological variability within these species. Detailed comments for the authors have been provided in the attached PDF file.

1. *This study has several limitations that should be acknowledged. One limitation of this study is the potential variability of results across different laboratory settings. Although standardized protocols were followed, differences in equipment, operator handling, reagent batches, and culture conditions may influence DNA yield, fragment length, and sequencing performance. Such inter-laboratory variability could affect the reproducibility of results obtained with the evaluated extraction kits. Therefore, while the BT MasterPure kit demonstrated superior performance under the conditions tested, its relative advantage should be confirmed in additional laboratory environments and across a wider range of strains.*

We thank the reviewer for this relevant comment. We acknowledge that this study was conducted in a single laboratory by a single team, which represents a limitation for the absolute generalization of the relative kit performances reported here. Nevertheless, we sought to limit technical variability by using standardized protocols and reagents from the same batch for all extractions. We also note that the performance of the BT MasterPure kit was consistent across two independent sequencing runs and five distinct strains (one reference strain and several clinical/environmental isolates), providing an initial indication of robustness. We have added a sentence to the Discussion (limitations paragraph Line 498–501) specifying that multicentric inter-laboratory studies would be needed to confirm the generalizability of these findings.

2. *First, the number of strains evaluated was limited, and only reference strains were included. Although these strains allow for controlled comparisons, they do not fully represent the genetic and phenotypic diversity observed among clinical isolates. The inclusion of clinical strains would have provided a more comprehensive evaluation of the performance of the extraction kits under conditions relevant to routine diagnostic and epidemiological investigations.*

We would like to clarify that the study did not rely solely on reference strains. Of the seven strains included, only two (*C. difficile* CD1 and *C. perfringens* CP1) are reference strains (DSM/ATCC). The remaining five strains are clinical or environmental isolates: *C. difficile* CD2 (sewage sludge) and CD3 (human stool, metronidazole-resistant), and *C. perfringens* CP2 (treated wastewater effluent), CP3 (bovine isolate, toxinotype E) and CP4 (human stool isolate, toxinotype G), as detailed in Table 1. Several of these isolates were specifically selected for the presence of plasmid-encoded toxin or antimicrobial resistance genes, precisely to assess the kits performance under conditions relevant to diagnostic and epidemiological

surveillance, as suggested by the reviewer. We have revised the manuscript (results and abstract) to make this distinction clearer to the reader (Lines 47 and 216–218).

3. *Furthermore, this study focused specifically on Clostridioides difficile and Clostridium perfringens. While these are clinically relevant spore-forming Gram-positive bacteria, the applicability of these findings to other Gram- positive organisms remains to be determined and warrants further investigation. It should also be noted that this study specifically focuses on Clostridioides difficile and Clostridium perfringens. While these organisms are clinically important spore-forming Gram-positive bacteria, the findings may not be directly transferable to other Gram-positive species with different cell wall structures or physiological characteristics. Further studies are needed to evaluate whether the observed performance of the DNA extraction methods is consistent across a broader range of clinically relevant Gram-positive bacteria.*

We agree with the reviewer that this study is limited in scope to two Gram-positive, spore-forming species, *C. difficile* and *C. perfringens*. These two species were specifically selected because they share the thick peptidoglycan cell wall and spore-forming physiology that make high-molecular-weight DNA extraction particularly challenging, and because of their direct clinical and public health relevance. We agree that extrapolation of these findings to other Gram-positive bacteria, particularly non-spore-forming species or species with markedly different cell wall composition, should be done with caution.

4. *In addition, The relatively small number of strains analyzed in this study limits the robustness of the conclusions, as it may not adequately capture the biological variability within these species and may affect the generalizability of the observed differences between extraction methods.*

We agree that the limited number of strains is the main limitation of this study, as acknowledged in the Discussion. We would like to note, however, that the number of strains used in this study is consistent with, and in several cases exceeds, that of comparable published studies evaluating sequencing methods or DNA extraction approaches for ONT sequencing. For instance, Berbers et al. (2020) characterized AMR genes on plasmids using long-read sequencing on a single bacterial strain; Eagle et al. (2023) evaluated five commercial DNA extraction kits for Nanopore sequencing using a single Salmonella isolate; Thornval et al. (2025) evaluated four extraction kits across four bacterial strains; and Gand et al. (2023) compared six DNA extraction methods using two bacterial species. Even Zidane et al. (2024), already cited in this manuscript, evaluated ONT R10 genotyping performance on three bacterial species without extensive strain replication. Similarly, Wick et al. (2017), also cited in this manuscript, used 12 isolates of a single species to evaluate a multiplexed MinION sequencing approach. Taken together, these examples suggest that the strain numbers used in the present study are consistent with the current standard for this type of methodological evaluation, and that the conclusions drawn here, while requiring confirmation in future studies with larger and more diverse strain panels, are well-supported by the available data.

REVIEWER #2

The study by Mrozinski et al. concerns the evaluation of three commercial kits to generate high-quality high-molecular weight DNA for optimal Oxford Nanopore sequencing of Clostridioides difficile and Clostridium perfringens.

The aim of the study is interesting, but the scope is limited:

1. *The number of strains sequenced is not sufficient to allow broad generalization of the results or to reach solid conclusions. This limitation is also acknowledged by the authors in line 476. Nanopore platforms allow the sequencing of tens of strains, or even more.*

We thank the reviewer for this comment and agree that the number of strains tested limits the extent to which broad conclusions can be drawn, as acknowledged in the Discussion. We would like to clarify that the primary objective of this study was a controlled comparison of extraction kit performance rather than a strain-diversity survey; the strain panel was therefore sized to allow direct, paired comparisons of the three kits on the same strains under R1, and confirmatory testing under R2. We also note that the number of strains used in this study is consistent with comparable published studies evaluating DNA extraction or sequencing methods for ONT sequencing, as detailed in our response to Reviewer #1, Comment 4 above (Berbers et al., 2020; Eagle et al., 2023; Gand et al., 2023; Thornval et al., 2025; Wick et al., 2017; Zidane et al., 2024). We agree that extending this comparison to a larger and more clinically and environmentally diverse strain collection represents an important direction for future work, and we have slightly expanded the limitations paragraph to make this point explicit.

2. *The study lacks extraction and sequencing replicates.*

We agree with the reviewer that this study did not include technical or biological replicates for each strain/kit combination, and we acknowledge this as a limitation of the present design. However, we would like to point out that, in the context of this study, the three extraction kits applied to the same strains can themselves be considered as a form of technical validation: all three kits consistently produced high-quality genomes for both species, with identical sequence types, antimicrobial resistance gene profiles, and toxin gene content, and with highly concordant cgMLST profiles regardless of the kit used. This cross-kit consistency provides strong evidence that the genomic data generated are robust and reproducible. Technical replication would have been particularly critical in the case of poor or inconsistent DNA quality (as assessed by Nanodrop, Qubit, and TapeStation) or poor sequencing data quality, but the uniformly high quality metrics observed across all kits and strains, both at the DNA extraction and sequencing levels, make this a less pressing concern in the present dataset. As a further indication of robustness, the superior performance of the BT MasterPure kit was consistent in direction across multiple independent strains (CD1, CD2 in R1; CD3, CP3, CP4 in R2) and across two separate sequencing runs performed at different times. We have added this limitation explicitly to the Discussion and recommend that future studies incorporate extraction and/or sequencing replicates to formally quantify technical variability (Lines 491–497).

3. *The use of commercial kits also carries the risk that, if the kits are modified by the manufacturers or replaced by newer versions, the findings of this study become obsolete.*

We agree with the reviewer that, as with any evaluation of commercial products, the specific kits tested here may be modified or discontinued by manufacturers over time. We note, however, that the use of standardized, commercially available kits is common practice in the field and represents the most practical and reproducible approach for laboratories seeking to implement validated extraction protocols, compared with in-house or custom-developed methods whose composition and performance can be more difficult to standardize and reproduce across laboratories. We have added the versions and dates of the manufacturer protocols (Lines 122–125).

4. *The authors tested the kits using only one long-read sequencing technology, which further limits the importance of the findings. The authors should also have checked the results using the PacBio platform to assess the broader applicability of these commercial kits.*

We would like to clarify that this study was specifically designed and scoped to evaluate DNA extraction kits for Oxford Nanopore sequencing, as reflected in the title and stated objective of the manuscript, a comparison including PacBio sequencing was beyond the scope and resources of the present study. We agree, however, that this represents an interesting direction for future work.

REVIEWER #3

The experiments presented in the manuscript compare 3 commercial DNA extraction kits for obtaining high molecular weight DNA and complete plasmids from Gram positive spore-forming species (3 strains of Clostridioides difficile and 3 strains of Clostridium perfringens). The metrics include DNA yield, fragment size and nanopore sequencing performance. The authors use a citation to pinpoint DNA yield and fragment size as the major limiting factor, due to the mechanical disruption techniques currently employed for cell lysis. The use of enzymatic lysis represents a return to classic methods for obtaining high molecular weight DNA. The reliability of the current methods has not been assessed (or at least there are no references given).

We thank the reviewer for this comment. The statement regarding the limitations of mechanical disruption methods was supported by Metcalf and Weese (2012), who reported fragmented and reduced-quality DNA when comparing extraction methods including mechanical lysis for *C. difficile*. This is also consistent with general best-practice guidance for ONT sequencing, which recommends minimizing mechanical shearing and favoring enzymatic lysis approaches to preserve HMW DNA.

According to the introduction, the study aimed to identify the method with the highest yield and the best read quality which most efficiently supported complete genome assembly. Only yield and fragment size differed among the methods, as they all resulted in successful genome assembly with genome completeness over 98%, even though coverage varied from 36X to over 100X, depending on the DNA source.

We thank the reviewer for this observation, with which we agree. As shown in Table 2, all three kits enabled successful, circularized genome assemblies with BUSCO completeness above 97.8% for both species, despite coverage ranging from 36X to 118X for *C. difficile*. As discussed in the manuscript, even the lowest coverage obtained (36X) was sufficient for complete genome circularization and reliable gene detection. We clarify that the main practical advantage of the BT MasterPure kit lies in providing a greater margin of sequencing coverage, combined with its lower cost and ease of use, rather than in being strictly necessary to achieve a usable assembly. We have added a few details in the revised abstract.

Since the plasmid complement of the strains was not determined experimentally, it is difficult to ascertain whether the sequencing data adequately represents the actual plasmid complement of the strains tested. However, since there were few missing genes, (98.5% to 100% of genes identified), coverage of all plasmid genes appears to be inferred rather than observed.

We agree with the reviewer that, with the exception of the *C. difficile* CD3 plasmid (pCD-METRO), whose presence had been independently confirmed by PCR prior to sequencing, plasmid characterization in this study relied on sequencing based evidence rather than direct experimental confirmation of plasmid structure. We note, however, that the presence of plasmids in CP3 and CP4 was not solely inferred from the sequencing data, it is independently supported by their toxinotype (E and G respectively), since the corresponding toxin genes (*cpe*/*iap*/*ibp* and *netB*) are known to be exclusively plasmid-encoded in *C. perfringens*. The sequencing-based evidence is therefore strongest for the fully circularized CP3 plasmid (pCPPB-1, 99.9% identity), and comparatively more limited for the two non-circularized CP4 plasmids, where full structural resolution could not be confirmed. We have clarified in the Discussion (Lines 469–477) that the non-circularization of these plasmids most likely reflects the presence of repetitive elements and can prevent assembly algorithms from unambiguously resolving the circular junction despite adequate sequencing coverage, rather than an absence or incomplete recovery of the plasmid sequences themselves.

Specific comments and suggestions:

The manuscript could be streamlined, considering the data presented. Methods should not be repeated in the results section (ex L212, L228, L299). The results section could be shortened by not repeating data presented in tables and summarizing the interpretation of the results.

We have revised the Results section to remove repetitions of methodological details already described in the Methods section.

Extra sentences can be eliminated that present figures, which can be cited in parentheses at the end of the sentence. Example: L116, the figure could be cited in parentheses. However, the experimental design is simple enough that Figure 1 can be eliminated, since the text repeats the two experiments.

We thank the reviewer for this suggestion. We appreciate that the text is sufficiently explicit to describe the experimental design, however we believe that Figure 1 provides added value by offering a clear visual overview of the two run workflow, which can otherwise be difficult to follow when described in text alone. Extra sentences were removed (Line 118).

Figures 2 and 4 should be reduced in size while maintaining image resolution.

The size of Figures 2 and 4 as displayed may reflect a formatting artifact of the submission process rather than the intended final layout. We are confident that the figures will be appropriately sized in the final typeset version of the manuscript.

The term "filtration" in English refers to the physical method of separation, while the correct term for bioinformatics is "filtering" of data. This should be changed throughout the text and figures.

The reviewer is correct. We have replaced "filtration" with "filtering" throughout the manuscript text, figure and figure legends to accurately reflect the bioinformatic nature of this step.

L438: The authors may =have intended to refer to "germination" of spores, not "sporulation", given the growth conditions used.

We thank the reviewer for this comment. However, we would like to clarify that the term "sporulation" at Line 442 is intentional and scientifically accurate in this context. The sentence refers to the difficulty of inducing spore formation (sporulation) in *C. perfringens* under *in vitro* conditions, which implies that the cultures used in this study likely consisted predominantly of vegetative cells rather than spores. This is precisely the argument used to explain why DNA extraction from *C. perfringens* was generally less challenging than from *C. difficile*, where spores are well known to be present in overnight cultures.

L466: This sentence should be inverted to accurately reflect the implications of the study. The results do not include surveillance facilitation, so the sentence should focus on future surveillance that could be facilitated by using Oxford Nanopore sequencing coupled with enzymatic lysis and HMW DNA extraction kits. In addition, the authors should state what are the current problems with C. difficile and C. perfringens surveillance (is it plasmid detection, emerging resistances, detection of hypervirulent clones or outbreak detection?). Some background would be useful to support the advantages of improving long read sequencing.

We agree with the reviewer that the sentence at Line 478 was misleadingly phrased. We have revised it to focus on the future surveillance potential enabled by this methodology rather than implying that surveillance facilitation is a direct result of this study. We have also added brief context on the specific challenges in *C. difficile* and *C. perfringens* surveillance that long-read sequencing coupled with HMW DNA extraction could

help address, including the detection of plasmid-encoded resistance genes (such as pCD-METRO), the characterization of toxin-encoding plasmids, and the tracking of emerging resistances and hypervirulent clones.

Re: Spectrum02407-26R1 (Comparative evaluation of three high-molecular-weight DNA extraction methods for optimal Oxford Nanopore long-read sequencing of the Gram-positive spore-forming Clostridioides difficile and Clostridium perfringens)

Dear Dr. Edward Topp:

Your manuscript has been accepted, and I am forwarding it to the ASM production staff for publication. Your paper will first be checked to make sure all elements meet the technical requirements. ASM staff will contact you if anything needs to be revised before copyediting and production can begin. Otherwise, you will be notified when your proofs are ready to be viewed.

Data Availability: ASM policy requires that data be available to the public upon online posting of the article, so please verify all links to sequence records, if present, and make sure that each number retrieves the full record of the data. If a new accession number is not linked or a link is broken, provide production staff with the correct URL for the record. If the accession numbers for new data are not publicly accessible before the expected online posting of the article, publication may be delayed; please contact ASM production staff immediately with the expected release date.

Publication Fees: For information on publication fees and which article types have charges, please visit our website. We have partnered with Copyright Clearance Center (CCC) to collect author charges. If fees apply to your paper, you will receive a message from no-reply@copyright.com with further instructions. For questions related to paying charges through RightsLink, please contact CCC at ASM_Support@copyright.com or toll free at +1-877-622-5543. CCC makes every attempt to respond to all emails within 24 hours.

ASM Membership: Corresponding authors may join or renew ASM membership to obtain discounts on publication fees. Need to upgrade your membership level? Please contact Customer Service at Service@asmusa.org.

Embargo Policy: A press release may be issued as soon as the manuscript is posted on the Spectrum Latest Articles webpage. The corresponding author will receive an email with the subject line "ASM Journals Author Services Notification" when the article is available online.

Cover Image Submissions: If you would like to submit a potential Cover Image, please email a file and a short legend to spectrum@asmusa.org. Please note that we can only consider images that (i) the authors created or own and (ii) have not been previously published. By submitting, you agree that the image can be used under the same terms as the published article. Image File requirements: TIF/EPS, 7.5 inches wide by 8.25 inches tall (at least 2,250 pixels wide by 2,475 pixels tall), minimum 300 dpi resolution (600 dpi preferred), RGB, and no figure elements, e.g., arrows or panel labels. The legend should be a short description of the image, 1-2 sentences recommended. Please use the ASM Journals Cover Image Sizing Tool (https://journals.asm.org/pb-assets/html/asm-cover-sizing-tool.html) to ensure that your proposed cover image meets our size requirements.

The ASM Journals program strives for constant improvement in our submission and publication process. Please tell us how we can improve your experience by taking this quick Author Survey.

Thank you for submitting your paper to Spectrum.

Sincerely,
Erik Hom
Editor
Microbiology Spectrum
