# Supplementary material for: Comparative evaluation of three high-molecular-weight DNA extraction kits for Oxford Nanopore sequencing of Clostridioides difficile and Clostridium perfringens
Source: Microbiol Spectr. 2026 Sep 11;14(10):e02407-26. doi: 10.1128/spectrum.02407-26 (PMC13688846; doi:10.1128/spectrum.02407-26)
Supplement: Supplementary file 2 [file spectrum.02407-26-s0002.docx]

**Supplementary Table 1. Antimicrobial resistance genes and toxin genes identified in genomes of *C. difficile* (CD1, CD2) and *C. perfringens* (CP1, CP2) strains.** Screening was performed using ABRicate v.1.0.1 with the following databases: VFDB, NCBI AMRFinderPlus, and MEGARES. Detection of the netB toxin gene was performed using BLAST v.2.16.0 against the reference sequence EU143239.1. For each gene, presence (black), partial presence (light black) or absence (white) is indicated for each strain and extraction kit.

**Supplementary Figure 1. Mean Phred score distribution obtained before and after filtration for the four different strains sequenced in Experiment 1.** Distributions were determined for the two *C. difficile* (CD1, CD2) and the two *C. perfringens* (CP1, CP2) strains and for the three extraction kits. Global differences across kits were assessed using Kruskal-Wallis tests (p-value indicated in each panel title). Pairwise comparisons were performed using Mann-Whitney U tests on 1,000 balanced random subsamples; effect sizes (r) and significance levels (*** p < 0.001, ** p < 0.01) are displayed above each comparison bracket, after Benjamini-Hochberg correction for multiple comparisons. Violin shapes were trimmed to observed data range. Promega HMW: Promega Wizard High Molecular Weight DNA Extraction Kit; BT MasterPure: Biosearch Technologies MasterPure Complete DNA and RNA Purification Kit; NEB HMW: New England Biolabs Monarch High Molecular Weight DNA Extraction Kit for Tissue.
